# Acetaminophen changes the RNA m^6^A levels and m^6^A-related proteins expression in IL-1β-treated chondrocyte cells

**DOI:** 10.1186/s12860-022-00444-3

**Published:** 2022-10-27

**Authors:** Jie Gao, Yan Li, Zijin Liu, Dong Wang, Huawu Zhang

**Affiliations:** Department of Joint Surgery, Shandong Second Provincial General Hospital, No.4 Duanxing West Road, Huaiyin District, Jinan, 250022 Shandong People’s Republic of China

**Keywords:** Acetaminophen, Chondrocyte cell, Interleukin-1β, RNA N^6^-methyladenosine modification, ALKBH5, Inflammatory factors, Extracellular matrix

## Abstract

**Background:**

Acetaminophen is commonly recommended for the early analgesia of osteoarthritis. However, the molecular mechanism by which it acts remains unknown. The aim of this study is to investigate the effect of acetaminophen on inflammation and extracellular matrix degradation in human chondrocytes, and the possible molecular mechanisms involved in its effect.

**Methods:**

The normal chondrocyte cell line C28/I2 was treated with interleukin-1β to mimic the inflammatory state. Acetaminophen and the methylation inhibitor (cycloleucine) were used to treat interleukin-1β-induced C28/I2 cells. The expression of RNA N^6^-methyladenosine -related proteins was detected by RT-qPCR and western blot. The total RNA N^6^-methyladenosine level was measured by dot blot analysis and enzyme linked immunosorbent assay. The levels of interleukin-6, interleukin-8 and anti-tumor necrosis factor-α were measured by enzyme linked immunosorbent assay. The extracellular matrix synthesis and degradation were examined by western blot.

**Results:**

After interleukin-1β stimulated C28/I2 cells, the intracellular RNA N^6^-methyladenosine level increased, and the expression of regulatory proteins also changed, mainly including the increased expression of methyltransferase like 3 and the downregulated expression of AlkB family member 5. The use of cycloleucine inhibited interleukin-1β-induced inflammation and extracellular matrix degradation by inhibiting RNA N^6^-methyladenosine modification. In contrast, acetaminophen treatment counteracted interleukin-1β-induced changes in RNA N^6^-methyladenosine levels and regulatory protein expression. Furthermore, acetaminophen treatment of interleukin-1β-induced C28/I2 cells inhibited the secretion of interleukin-6, interleukin-8 and anti-tumor necrosis factor-α, down-regulated the expression of matrix metalloproteinase-13 and Collagen X, and up-regulated the expression of collagen II and aggrecan. In addition, AlkB family member 5 overexpression activated interleukin-1β-induced chondrocyte viability and suppressed inflammation and extracellular matrix degradation.

**Conclusion:**

Acetaminophen affects inflammatory factors secretion and extracellular matrix synthesis of human chondrocytes by regulating RNA N^6^-methyladenosine level and N^6^-methyladenosine-related protein expression.

**Graphical abstract:**

Stimulation of the normal chondrocyte cell line C28/I2 with the cytokine IL-1β (10 μM) mimics the inflammatory state in vitro. Acetaminophen (Ace, 50 μg/mL) changes the m^6^A related proteins expression and the total RNA m^6^A levels in IL-1β-treated chondrocyte cells. Furthermore, regulation of RNA m^6^A levels (by methylation inhibitor Cyc and/or Ace) affects IL-1β-induced inflammatory cytokines secretion and extracellular matrix synthesis in C28/I2 cells.
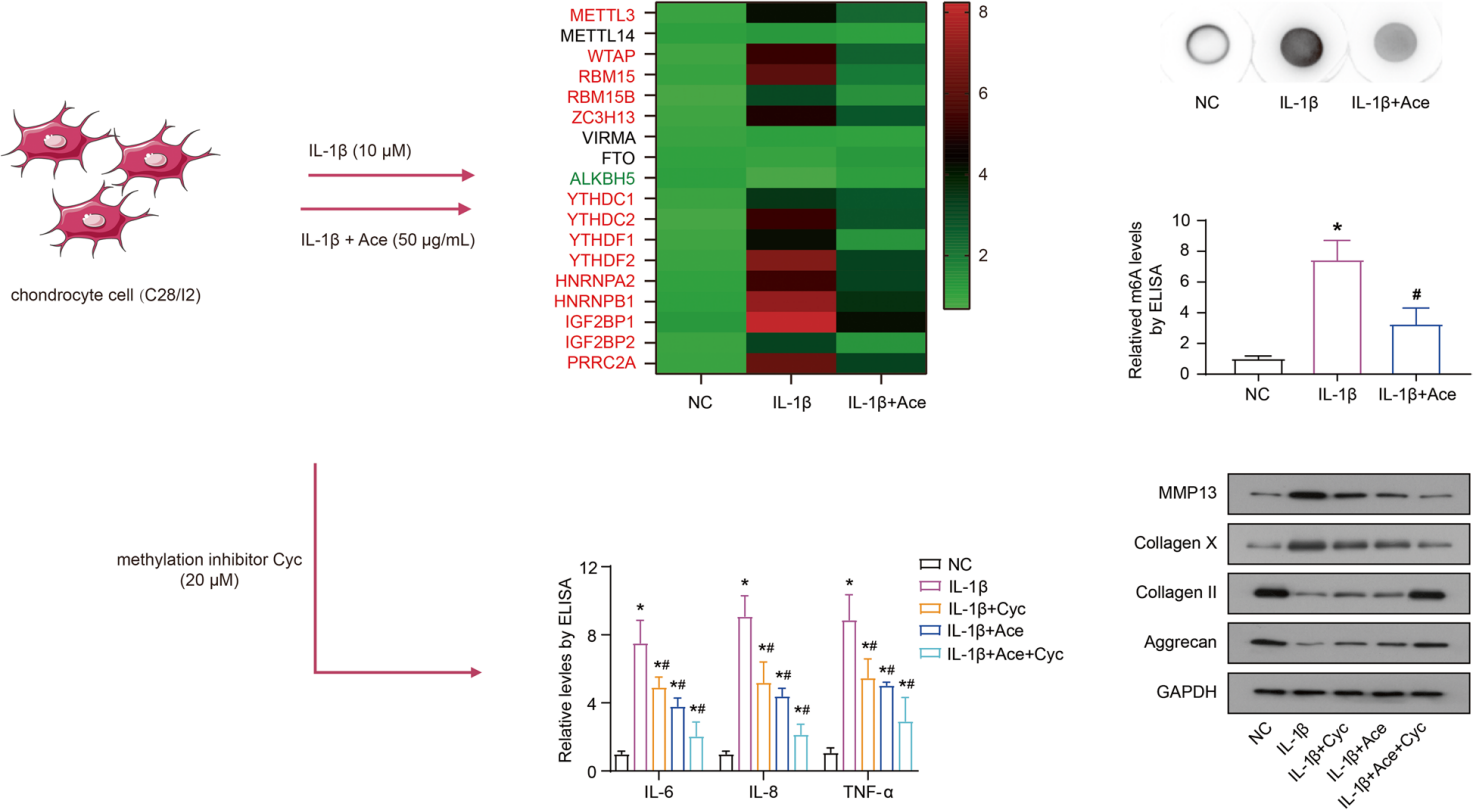

**Supplementary Information:**

The online version contains supplementary material available at 10.1186/s12860-022-00444-3.

## Background

Osteoarthritis (OA) is a pervasive degenerative and chronic inflammatory joint disease, and one of the top three causes of disability worldwide, affecting more than 250 million people worldwide [1, 2]. Due to aging and an increase in the obese/overweight population, OA will become more prevalent. The United Nations estimates that by 2050, approximately 15–20% of the population will suffer from OA, of which one third will be unable to perform daily activities [3]. Research on OA needs more attention.

At present, the clinical therapy of OA includes non-pharmacological, pharmacological and surgical treatments, which are used in combination when necessary. But these approaches are designed to relieve pain and slow disease progression, and do not reverse or actually treat OA. Non-drug therapy refers to lifestyle changes that promote healthy body composition through changes in diet, exercise, weight loss, and more. Nonpharmacological options are often underutilized. Pharmacological options have not improved significantly over the past few decades. Pharmacological treatment in the initial stage of the disease mainly includes acetaminophen (Ace, paracetamol), non-steroidal anti-inflammatory drugs (NSAIDs), and opioids. However, NSAIDs may cause gastrointestinal and cardiovascular complications. Opioids have addictive limitations. As OA worsens, intra-articular corticosteroids can be administered to suppress pain and inflammation, although their effects are often short-term. Most corticosteroids are hydrophobic, have short half-lives, and have insufficient retention time in joints. However, long-term use of corticosteroids should be avoided, with adverse effects including immunosuppression, osteoporosis, and diabetes. Furthermore, the role of nutritional supplements such as chondroitin sulfate in improving OA pain and inflammation is controversial [4]. Joint replacement surgery is the last option and is more expensive. In addition, the new development directions of pharmaceutical companies mainly include disease-modifying OA drugs, nano-drugs, etc., which have not yet shown good efficacy [5]. In general, the clinical treatment of OA has not progressed significantly in the past few decades, and a breakthrough is urgently needed.

Ace was first synthesized in 1878 from its precursor phenacetin. In the 1950s, Ace was marketed as a safer alternative to phenacetin, which was found to be nephrotoxic and potentially carcinogenic. In the 1980s, Ace began to gradually replace aspirin as the most widely used over-the-counter analgesic. At present, Ace is still one of the most commonly used analgesics and antipyretics in the world, and is included in the World Health Organization’s Essential Medicine List [2, 6]. Due to the belief in its relative safety and the lack of suitable alternatives, especially considering the safety of NSAIDs and opioids, Ace is recommended as first-line therapy for chronic diseases such as OA and low back pain [7, 8]. At present, the mechanism of action of Ace is not fully understood, but may involve cyclooxygenase-2 inhibition [9]. Therefore, it is necessary to explore the molecular mechanism of Ace’s effect on OA.

RNA methylation is necessary to maintain RNA metabolism and function, and participates in the entire process of human development and disease [10]. N^6^-methyladenosine (m^6^A) accounts for 80% of RNA methylation modifications, and is a common RNA modification in eukaryotes. m^6^A is installed by the m^6^A methyltransferase complex (METTL3 and METTL14) and its cofactors (WTAP, RBM15/15B, ZC3H13 and VIRMA), removed by FTO and ALKBH5 [11], and recognized by RNA binding proteins to perform specific biological functions, including YTHDC1/2, YTHDF1/2/3, HNRNPA2/B1, IGF2BP1/2/3 and Prrc2a [12–14]. Recent study shows that m^6^A methylation levels and METTL3 expression are significantly elevated in degenerative human cartilage tissue [15]. Changes in RNA m^6^A modification of various genes regulate their expression levels and participate in the biological processes involved in OA such as extracellular matrix (ECM) synthesis by chondrocytes [15–18].

In this study, the human chondrocyte cell line C28/I2 treated with interleukin (IL)-1β was used as the research object, to analyze the effect of Ace on the RNA m^6^A modification in C28/I2 cells treated with IL-1β, and whether it affects inflammatory factors secretion and ECM synthesis by regulating RNA m^6^A modification. However, this study did not reveal the specific molecular mechanism by which Ace regulates the differential expression of RNA m^6^A modification-related proteins.

## Methods

### Cell culture and treatment

Human normal chondrocyte cell line C28/I2 was purchased from Beijing Beina Chuanglian biotechnology institute (Beijing, China) and maintained in DMEM/F12 medium (1:1 ratio) supplemented with 10% (v/v) FBS (A31608, GIBCO) at 37 °C and 5% CO_2_. IL-1β (10 μM) (SRP3083), Ace (50 μg/mL) and cycloleucine (A48105) (Cyc, 20 μM) were purchased from Sigma Aldrich and used for treat cells for 12 h. The coding sequence of ALKBH5 was synthesized by Genechem (Shanghai, China), cloned into pcDNA3.1 expression vector, and transfected into the cells using Lipofectamine 2000 reagent (11668–019, Invitrogen).

### Quantitative RT-PCR

The cells were extracted with Trizol reagent for total RNA. After quantification by spectrophotometer, the equal amount of RNA was reverse transcribed into cDNA using PrimeScript RT Master Mix (CW2569M, CWBIO, China). The target genes were amplified using SYBR Green Real-time PCR Master Mix (CW0957H, CWBIO). The relative levels of gene expression (METTL3, METTL14, WTAP, RBM15/15B, ZC3H13, VIRMA, FTO, ALKBH5, YTHDC1/2, YTHDF1/2, HNRNPA2/B1, IGF2BP1/2/3 and Prrc2a) were calculated by the 2^-△Ct^ method, and the relative level of GADPH was as a control.

### Western blot

The cells were extracted with ice-cold RIPA lysis buffer (CW2333S, CWBIO) for total protein. After quantification with the BCA kit (CW0014S, CWBIO), the equal amount of total protein was separated by SDS-PAGE gel electrophoresis, and transferred to a PVDF membrane (ISEQ00010, Millipore, USA). After blocking with 5% skim milk, the membrane was incubated with primary antibody against ALKBH5 (1:2000, ab195377, Abcam), FTO (1:1000, ab126605, Abcam), Aggrecan (1:2000, ab36861, Abcam), Collagen X (1:2000, ab58632, Abcam), MMP13 (1:1000, ab39012, Abcam), or Collagen II (1:2000, ab188570, Abcam) at 4 °C overnight, and then incubated with horseradish-labeled secondary antibody (SA00001, PTG) at room temperature for 2 h. The membrane was treated with ECL (PK10002, PTG) for the band visualization.

### Total m^6^A level detection

For dot blot analysis, TRIzol reagent was used to isolate and purify total RNA. The total RNA was denatured under UV light and cross-linked with the optimized Amersham Hybond-N^+^ membrane. After cross-linking, the membrane was blocked with 5% skim milk, and incubated with anti-m^6^A antibody at 4 °C overnight. Immobilon Western Chemilum HRP substrate (Merck Millipore) was used for visualization. For ELISA, the EpiQuik m6A RNA Methylation Quantitative ELISA Kit (P-9005-96, Epigentek) was used according to the manufacturer’s instructions.

### ELISA for inflammatory factors detection

Levels of cytokines (IL-6, IL-8 and TNF-α) were assayed by ELISA (EH2IL6, KHC0081, KHC3011; Invitrogen) according to the manufacturer’s instructions.

### CCK-8 assay for cell viability analysis

After cells were transfected with pcDNA 3.1-ALKBH5 expression plasmid and treated with IL-1β for 12 h, 10 μL of CCK-8 reagent was added into each well and incubation for 2 h at 37 °C. The light absorbance value at 450 nm was measured with a microplate reader (BioTek Instruments, USA).

### Statistical analysis

Data was statistically analyzed by SPSS 20.0 and presented as mean ± SD. The significant difference between groups was analyzed by one way variance or Student’s t test. *P* < 0.05 was considerable significance.

## Results

### Ace changes the m^6^A levels and m^6^A related proteins expression in IL-1β-treated chondrocyte cells

**Fig. 1:**
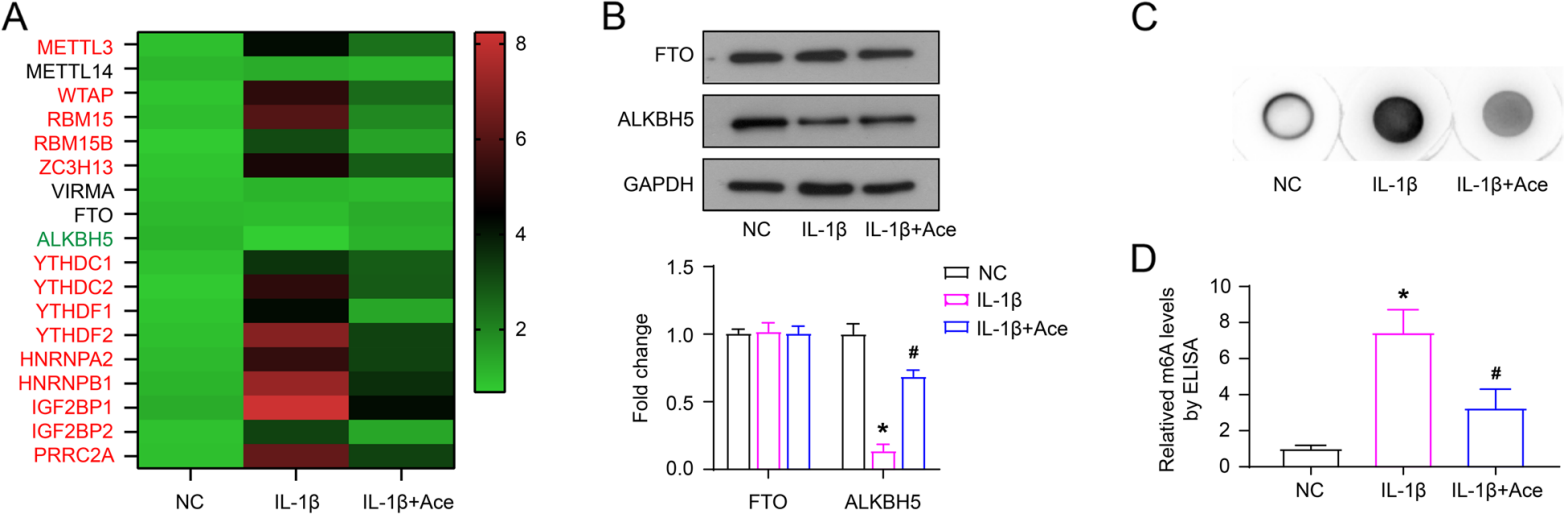
Ace changes the m^6^A levels and m^6^A related proteins expression in IL-1β-treated chondrocyte cells. The cytokine IL-1β (10 μM) and Ace (50 μg/mL) was used to treat C28/I2 cells. After incubating for 12 h, the mRNA expression levels of m^6^A-related proteins were detected by RT-qPCR (**A**), the protein levels of ALKBH5 and FTO were examined by western blot (**B**), the total m^6^A level of each group were measured by dot blot analysis (**C**) and ELISA (**D**)

The CTD database (http://ctdbase.org/) presumed that Ace application in OA would interact with these m^6^A-related proteins (Table 1). Stimulation of the normal chondrocyte cell line C28/I2 with the cytokine IL-1β (10 μM) mimics the inflammatory state in vitro. The inflammatory state was confirmed by ELISA detection of matrix metallopeptidase (MMP)-13 levels in the medium (Supplementary Fig. 1). IL-1β-induced C28/I2 cells were treated with Ace (50 μg/mL) (IL-1β + Ace). After 12 h of incubation, the mRNA levels of m^6^A-related proteins and the total m^6^A level of each group were detected (Fig. 1A-C). As shown in Fig. 1A, compared into NC group, the mRNA levels of METTL3, WTAP, RBM15, RBM15B, ZC3H13, YTHDC1, YTHDC2, YTHDF1, YTHDF2, HNRNPA2, HNRNPB1, IGF2BP1, IGF2BP2 and PRRC2A significantly increased in the IL-1β group, while the mRNA levels of those proteins in the IL-1β + Ace group obviously decreased compared with the IL-1β group. In addition, the mRNA and protein levels of ALKBH5 was reduced in IL-1β group compared into NC group, while it was recovered in IL-1β + Ace group (Fig. 1A and B). However, there was no significant change in the mRNA levels of METTL14, VIRMA and FTO (Fig. 1A and B). Furthermore, compared with NC group, the total m^6^A level increased in IL-1β group, and it was rescued by Ace treatment (Fig. 1C and D).

**Table 1:** Targets of Acetaminophen action related to m^6^A in Osteoarthritis

Drugs	Disease	Targets
Acetaminophen	Osteoarthritis	METTL3; WTAP; RBM15; RBM15B; ZC3H13
		ALKBH5
		YTHDC1; YTHDC2; YTHDF1; YTHDF2; HNRNPA2; HNRNPB1; IGF2BP1; IGF2BP2; PRRC2A

### Regulation of RNA m^6^A levels affects IL-1β-induced inflammatory cytokines secretion and ECM synthesis in C28/I2 cells

**Fig. 2:**
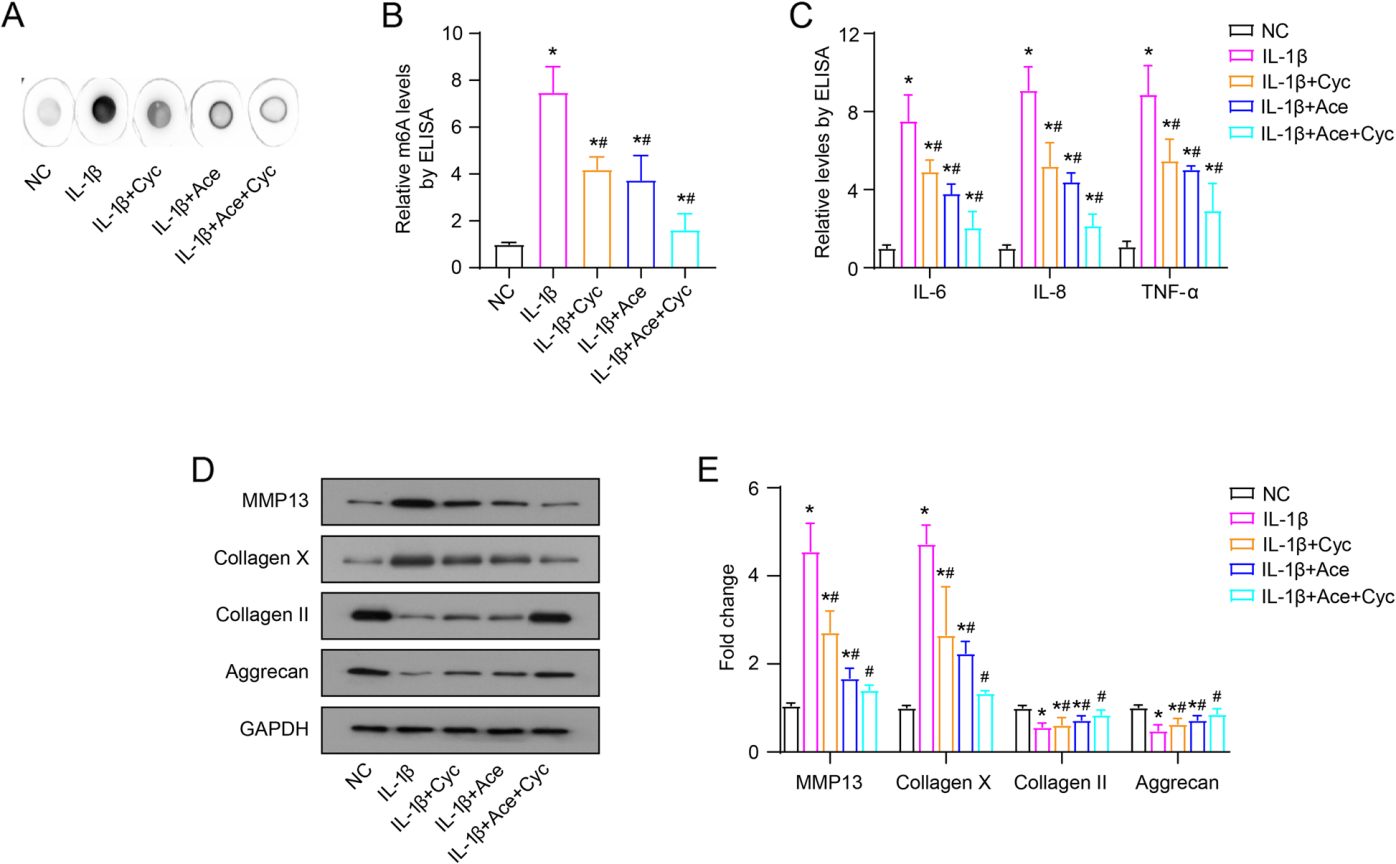
Cyc affects the secretion of inflammatory factors and the synthesis of ECM on the IL-1β-induced C28/I2 cells. Cyc (10 μM) treated the IL-1β-induced C28/I2 cells. After incubating for 12 h, the total m^6^A level of each group were measured by dot blot analysis (**A**) and ELISA (**B**), the levels of inflammatory factors (IL-6, IL-8 and TNF-α) were measured by ELISA (**C**), the protein levels of collagen were examined by western blot (**D** and **E**)

In view of the effect of IL-1β and/or Ace treatment on the m^6^A modification of C28/I2 cells, we performed validation experiments using the methylation inhibitor Cyc. As shown in Fig. 2A and B, Cyc (10 μM) treatment reduced the total m^6^A level of IL-1β-induced C28/I2 cells, and Cyc and Ace had a synergistic effect. Moreover, Cyc and Ace treatment alone reduced the inflammatory cytokines (IL-6, IL-8 and TNF-α) secretion, down-regulated MMP-13 and Collagen X expression, and up-regulated the expression of Collagen II and Aggrecan, which all regulated by IL-1β (Fig. 2C-E). These results suggest that regulation of RNA m^6^A levels affects IL-1β-induced inflammatory cytokines secretion and ECM synthesis in C28/I2 cells.

### Overexpression of ALKBH5 affects the viability, inflammatory factors secretion and ECM synthesis of IL-1β-induced C28/I2 cells

**Fig. 3:**
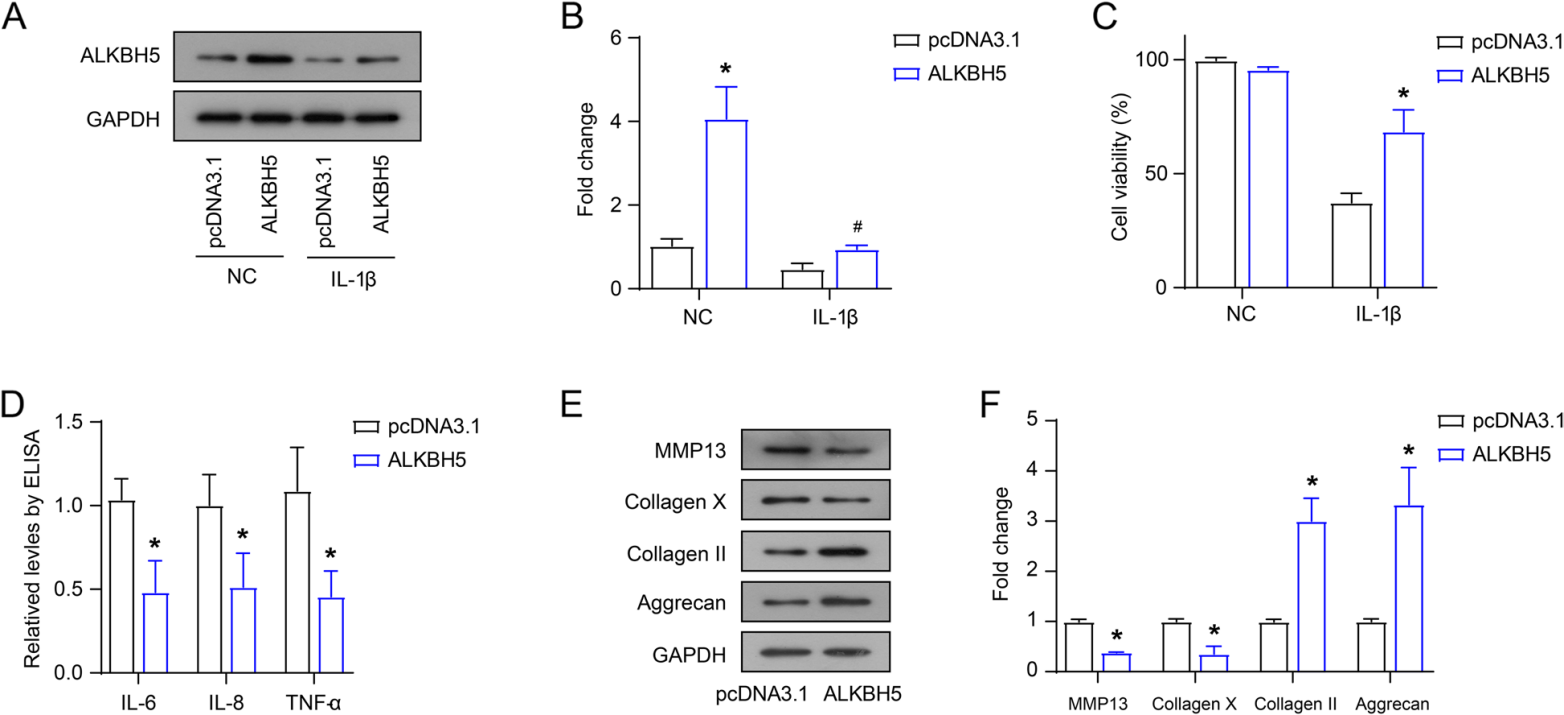
Overexpression of ALKBH5 affects the viability, inflammatory factors secretion and ECM synthesis of IL-1β-induced C28/I2 cells. After transfection of pcDNA 3.1-ALKBH5 expression plasmid, the protein expression of ALKBH5 in NC and IL-1β-induced C28/I2 cells were examined by western blot (**A** and **B**), the viability was detected by CCK-8 assay (**C**), the levels of inflammatory factors (IL-6, IL-8 and TNF-α) were measured by ELISA (**D**), the protein levels of collagen were examined by western blot (**E** and **F**)

In view of the unique role of ALKBH5 in the Ace treatment of IL-1β-induced C28/I2 cells, we tested the role of ALKBH5 overexpression on the viability, inflammatory factors secretion and ECM synthesis of IL-1β-induced C28/I2 cells. As shown in Fig. 3A and B, the transfection of pcDNA 3.1-ALKBH5 expression plasmid up-regulated the protein expression of ALKBH5 in NC and IL-1β-induced C28/I2 cells. Overexpression of ALKBH5 improved the viability of IL-1β-induced C28/I2 cells, and had no significant effect on the viability of NC cells (Fig. 3C). Furthermore, overexpression of ALKBH5 suppressed the secretion of inflammatory factors (IL-6, IL-8 and TNF-α), down-regulated the expression of MMP-13 and Collagen X, and up-regulated the expression of Collagen II and Aggrecan in IL-1β-induced C28/I2 cells (Fig. 3D-F).

## Discussion

In recent years, the benefits of Ace in chronic diseases have been questioned. Several randomized controlled trials found a statistically significant difference between Ace and placebo for OA pain, but moderate analgesic efficacy [19]. Concerns about the adverse effects of long-term use of Ace also increased during the same period, with major adverse effects including an increased risk of gastrointestinal bleeding and a small increase in systolic blood pressure [8, 20, 21]. However, in the face of no suitable alternative medicines, Ace is still widely recommended for the early analgesic treatment of OA, and is often prescribed [7, 22, 23]. In addition, the molecular mechanism of Ace’s application in OA treatment is still unknown. Therefore, it is necessary to explore the molecular and pharmacological mechanism of Ace’s effect on OA, which can provide a certain basis for the application guide of Ace.

OA is considered to be the degeneration of cartilage. One of its major pathogenesis is that pro-inflammatory cytokines activate catabolic enzymes (such as MMP13), which in turn damage cartilage and other intra-articular structures [24]. IL-1β plays a key role in OA progression. It can activate different signaling pathways, and induce the production of other inflammatory cytokines (such as IL-6, IL-8 and TNF-α) and MMPs, and induce cartilage degradation [25]. The combination leads to the progression of OA. The extracellular skeleton of adult articular cartilage is aggregated collagen, and collagen II is its major molecular component [26]. Collagen X is usually confined to the thin layer of calcified cartilage connecting articular cartilage and bone, and is expressed and deposited in OA lesions [27]. This study found that Ace treatment of IL-1β-induced C28/I2 cells can inhibit the secretion of IL-6, IL-8 and TNF-α, down-regulated the expression of MMP-13 and Collagen X, and up-regulated the expression of Collagen II and Aggrecan (Fig. 2). These results of these in vitro cell experiments demonstrate that Ace treatment can inhibit the inflammation response and ECM degradation of human chondrocytes, appear to suggest the pharmacological role of Ace in OA progression, but convincing animal and/or human studies are lacking.

Furthermore, recent reports indicate that the RNA m^6^A modification process is involved in the OA progression by regulating the mRNA stability and expression of multiple target genes [28, 29]. The results of this study showed that IL-1β stimulated C28/I2 cells with increased RNA m^6^A levels and changes in the expression of regulatory proteins, mainly including increased expression of METTL3 and down-regulated expression of ALKBH5 (Fig. 1). The use of the RNA m^6^A inhibitor Cyc also suggested a critical role of the RNA m^6^A modification process in the inflammation and ECM degradation of OA progression (Fig. 2). Consistent with our study, it has been reported that intracellular RNA m^6^A levels and METTL3 expression are increased after IL-1β stimulation of the chondrogenic progenitor cell line ATDC5 cells [30]. Significant increases in METTL3 expression and RNA m^6^A levels have also been reported in degenerative cartilage tissue [15]. In contrast, one study analyzes the expression patterns of 18 RNA m^6^A -modified proteins included in this study in 18 normal cartilage and 20 OA cartilage tissues [31]. The results of this study are not the same as ours. However, there are significant differences in the gender and age of the patients from which the samples are derived between the two groups [32]. Normal cartilages are obtained from 5 women and 13 men, with a mean age of 38 years (18–61 years), and samples are taken 24 hours after the death of the patients. The OA cartilage samples are obtained from 12 women and 8 men with a mean age of 66 years (52–82 years). As we all know, OA is a disease closely related to age and gender. This may be the main reason for the different results of the two studies. Crucially, the present study found that Ace treatment counteracted IL-1β-induced changes in RNA m^6^A levels and regulatory protein expression (Fig. 1).

Finally, as a methylase, METTL3 has certain studies in OA progression. However, as a major demethylase, the role of ALKBH5 in OA progression is understudied. This study demonstrates for the first time that ALKBH5 overexpression activates IL-1β-induced chondrocyte viability and inhibits inflammation and ECM degradation. However, also as a demethylase, FTO showed no significant changes under neither IL-1β nor Ace treatment. We lack research and explanation for this.

## Conclusions

First, the data from this study, as well as other research reports, suggest that differential expression of RNA m^6^A-related proteins and elevated levels of total RNA m^6^A are involved in OA progression. The total RNA m^6^A modification changes regulated the expression of downstream target genes, and their involvement in OA progression still requires further study. Whether it is possible to design drugs based on RNA m^6^A -related proteins and/or total RNA m^6^A levels to delay the OA progression also requires more basic research as data support. Furthermore, Ace treatment was able to affect the expression of RNA m^6^A-related proteins in chondrocytes as well as total RNA m^6^A levels. However, this study did not reveal the specific mechanism of Ace’s effect. Finally, Ace can inhibit the inflammatory response and ECM degradation in IL-1β-treated human chondrocytes in vitro, indicating the pharmacological effects of Ace. However, more convincing animal and/or human studies are needed.

## Supplementary Information

**Additional file 1: Supplementary Figure 1.** MMP-13 levels in the medium of IL-1β-induced C28/I2 cells detected by ELISA.

**Additional file 2: Supplementary Figure 2.** The original versions of Fig. 1B. **Supplementary Figure 3.** The original versions of Fig. 2D. **Supplementary Figure 4.** The original versions of Fig. 3A. **Supplementary Figure 5.** The original versions of Fig. 3E.

## Data Availability

All data generated or analysed during this study are included in this published article.

## Abbreviations

OA: Osteoarthritis
m^6^A: N^6^-methyladenosine
METTL3: Methyltransferase like 3
METTL14: Methyltransferase like 14
WTAP: Wilms tumor 1 associated protein
RBM15/15B: RNA binding motif protein 15/15B
ZC3H13: Zinc finger CCCH-type containing 13
VIRMA: Vir like m^6^A methyltransferase associated
FTO: Fat mass and obesity associated
ALKBH5: AlkB family member 5
YTHDC1/2: YTH domain containing 1/2
YTHDF1/2/3: YTH N(^6^)-methyladenosine RNA binding protein 1/2/3
HNRNPA2/B1: Heterogeneous nuclear ribonucleoprotein A2/B1
IGF2BP1/2/3: Insulin-like growth factor 2 mRNA binding protein 1/2/3
Prrc2a: Proline-rich coiled-coil 2A
ECM: Extracellular matrix
Cyc: Cycloleucine
IL: Interleukin
TNF-α: Tumor necrosis factor-α
MMP-13: Matrix metallopeptidase 13

## References

[1] Disease GBD, Injury I, Prevalence C (2018). Global, regional, and national incidence, prevalence, and years lived with disability for 354 diseases and injuries for 195 countries and territories, 1990-2017: a systematic analysis for the Global Burden of Disease Study 2017. Lancet..

[2] Vos T, Flaxman AD, Naghavi M, Lozano R, Michaud C, Ezzati M (2012). Years lived with disability (YLDs) for 1160 sequelae of 289 diseases and injuries 1990-2010: a systematic analysis for the Global Burden of Disease Study 2010. Lancet..

[3] Papathanasiou I, Anastasopoulou L, Tsezou A (2021). Cholesterol metabolism related genes in osteoarthritis. Bone..

[4] Colletti A, Cicero AFG. Nutraceutical approach to chronic osteoarthritis: from molecular research to clinical evidence. Int J Mol Sci. 2021;22(23). 10.3390/ijms222312920. 10.3390/ijms222312920 PMC8658017 34884724

[5] Wang Z, Wang S, Wang K, Wu X, Tu C, Gao C (2021). Stimuli-sensitive nanotherapies for the treatment of osteoarthritis. Macromol Biosci.

[6] Organization WH (2017). World Health Organization model list of essential medicines.

[7] Bruyere O, Cooper C, Pelletier JP, Branco J, Luisa Brandi M, Guillemin F (2014). An algorithm recommendation for the management of knee osteoarthritis in Europe and internationally: a report from a task force of the European Society for Clinical and Economic Aspects of Osteoporosis and Osteoarthritis (ESCEO). Semin Arthritis Rheum.

[8] McCrae JC, Morrison EE, MacIntyre IM, Dear JW, Webb DJ (2018). Long-term adverse effects of paracetamol - a review. Br J Clin Pharmacol.

[9] Aminoshariae A, Khan A (2015). Acetaminophen: old drug, new issues. J Endod.

[10] Covelo-Molares H, Bartosovic M, Vanacova S (2018). RNA methylation in nuclear pre-mRNA processing. Wiley Interdiscip Rev RNA.

[11] Huang Y, Yan J, Li Q, Li J, Gong S, Zhou H (2015). Meclofenamic acid selectively inhibits FTO demethylation of m6A over ALKBH5. Nucleic Acids Res.

[12] Liu N, Dai Q, Zheng G, He C, Parisien M, Pan T (2015). N(6)-methyladenosine-dependent RNA structural switches regulate RNA-protein interactions. Nature..

[13] Huang H, Weng H, Sun W, Qin X, Shi H, Wu H (2018). Recognition of RNA N(6)-methyladenosine by IGF2BP proteins enhances mRNA stability and translation. Nat Cell Biol.

[14] Wu R, Li A, Sun B, Sun JG, Zhang J, Zhang T (2019). A novel m(6)A reader Prrc2a controls oligodendroglial specification and myelination. Cell Res.

[15] Xiao L, Hu B, Ding B, Zhao Q, Liu C, Oner FC (2022). N(6)-methyladenosine RNA methyltransferase like 3 inhibits extracellular matrix synthesis of endplate chondrocytes by downregulating sex-determining region Y-Box transcription factor 9 expression under tension. Osteoarthr Cartil.

[16] Han J, Kong H, Wang X, Zhang XA. Novel insights into the interaction between N6-methyladenosine methylation and noncoding RNAs in musculoskeletal disorders. Cell Prolif. 2022:e13294. 10.1111/cpr.13294. 10.1111/cpr.13294 PMC9528765 35735243

[17] Ren J, Li Y, Wuermanbieke S, Hu S, Huang G (2022). N(6)-methyladenosine (m(6)A) methyltransferase METTL3-mediated LINC00680 accelerates osteoarthritis through m(6)A/SIRT1 manner. Cell Death Dis.

[18] Zhou H, Shen X, Yan C, Xiong W, Ma Z, Tan Z (2022). Extracellular vesicles derived from human umbilical cord mesenchymal stem cells alleviate osteoarthritis of the knee in mice model by interacting with METTL3 to reduce m6A of NLRP3 in macrophage. Stem Cell Res Ther.

[19] Saragiotto BT, Machado GC, Ferreira ML, Pinheiro MB, Abdel Shaheed C, Maher CG (2016). Paracetamol for low back pain. Cochrane Database Syst Rev.

[20] Jozwiak-Bebenista M, Nowak JZ (2014). Paracetamol: mechanism of action, applications and safety concern. Acta Pol Pharm.

[21] McLachlan AJ, Bath S, Naganathan V, Hilmer SN, Le Couteur DG, Gibson SJ (2011). Clinical pharmacology of analgesic medicines in older people: impact of frailty and cognitive impairment. Br J Clin Pharmacol.

[22] Bannuru RR, Osani MC, Vaysbrot EE, Arden NK, Bennell K, Bierma-Zeinstra SMA (2019). OARSI guidelines for the non-surgical management of knee, hip, and polyarticular osteoarthritis. Osteoarthr Cartil.

[23] Blieden M, Paramore LC, Shah D, Ben-Joseph R (2014). A perspective on the epidemiology of acetaminophen exposure and toxicity in the United States. Expert Rev Clin Pharmacol.

[24] Molnar V, Matisic V, Kodvanj I, Bjelica R, Jelec Z, Hudetz D, et al. Cytokines and chemokines involved in osteoarthritis pathogenesis. Int J Mol Sci. 2021;22(17). 10.3390/ijms22179208. 10.3390/ijms22179208 PMC8431625 34502117

[25] Min S, Wang C, Lu W, Xu Z, Shi D, Chen D (2017). Serum levels of the bone turnover markers dickkopf-1, osteoprotegerin, and TNF-alpha in knee osteoarthritis patients. Clin Rheumatol.

[26] Eyre D (2002). Collagen of articular cartilage. Arthritis Res.

[27] He Y, Siebuhr AS, Brandt-Hansen NU, Wang J, Su D, Zheng Q (2014). Type X collagen levels are elevated in serum from human osteoarthritis patients and associated with biomarkers of cartilage degradation and inflammation. BMC Musculoskelet Disord.

[28] Shi L, Hu H, Sun P, Li Z, Ji L, Liu S (2022). RPL38 knockdown inhibits the inflammation and apoptosis in chondrocytes through regulating METTL3-mediated SOCS2 m6A modification in osteoarthritis. Inflamm Res.

[29] Liu Y, Yang Y, Lin Y, Wei B, Hu X, Xu L, et al. N(6) -methyladenosine-modified circRNA RERE modulates osteoarthritis by regulating beta-catenin ubiquitination and degradation. Cell Prolif. 2022:e13297. 10.1111/cpr.13297. 10.1111/cpr.13297 PMC9816929 35733354

[30] Liu Q, Li M, Jiang L, Jiang R, Fu B (2019). METTL3 promotes experimental osteoarthritis development by regulating inflammatory response and apoptosis in chondrocyte. Biochem Biophys Res Commun.

[31] Duan Y, Yu C, Yan M, Ouyang Y, Ni S (2022). m6A regulator-mediated RNA methylation modification patterns regulate the immune microenvironment in osteoarthritis. Front Genet.

[32] Fisch KM, Gamini R, Alvarez-Garcia O, Akagi R, Saito M, Muramatsu Y (2018). Identification of transcription factors responsible for dysregulated networks in human osteoarthritis cartilage by global gene expression analysis. Osteoarthr Cartil.

