# Supplementary figures and images for: Acetaminophen changes the RNA m6A levels and m6A-related proteins expression in IL-1β-treated chondrocyte cells
Source: BMC Mol Cell Biol. 2022 Oct 27;23:45. doi: 10.1186/s12860-022-00444-3 (PMC9609262; doi:10.1186/s12860-022-00444-3)

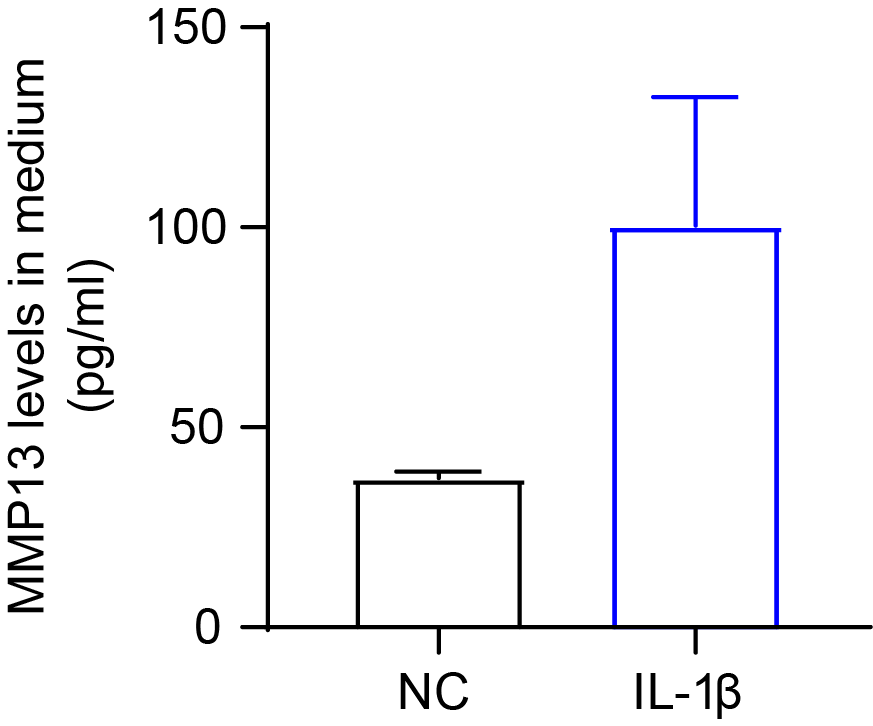

Supplement: Supplementary file 1 — Additional file 1: Supplementary Figure 1. MMP-13 levels in the medium of IL-1β-induced C28/I2 cells detected by ELISA. [file 12860_2022_444_MOESM1_ESM.tif]
