# Supplementary material for: Acetaminophen changes the RNA m6A levels and m6A-related proteins expression in IL-1β-treated chondrocyte cells
Source: BMC Mol Cell Biol. 2022 Oct 27;23:45. doi: 10.1186/s12860-022-00444-3 (PMC9609262; doi:10.1186/s12860-022-00444-3)

Supplementary Figure 2: The original versions of Figure 1B.

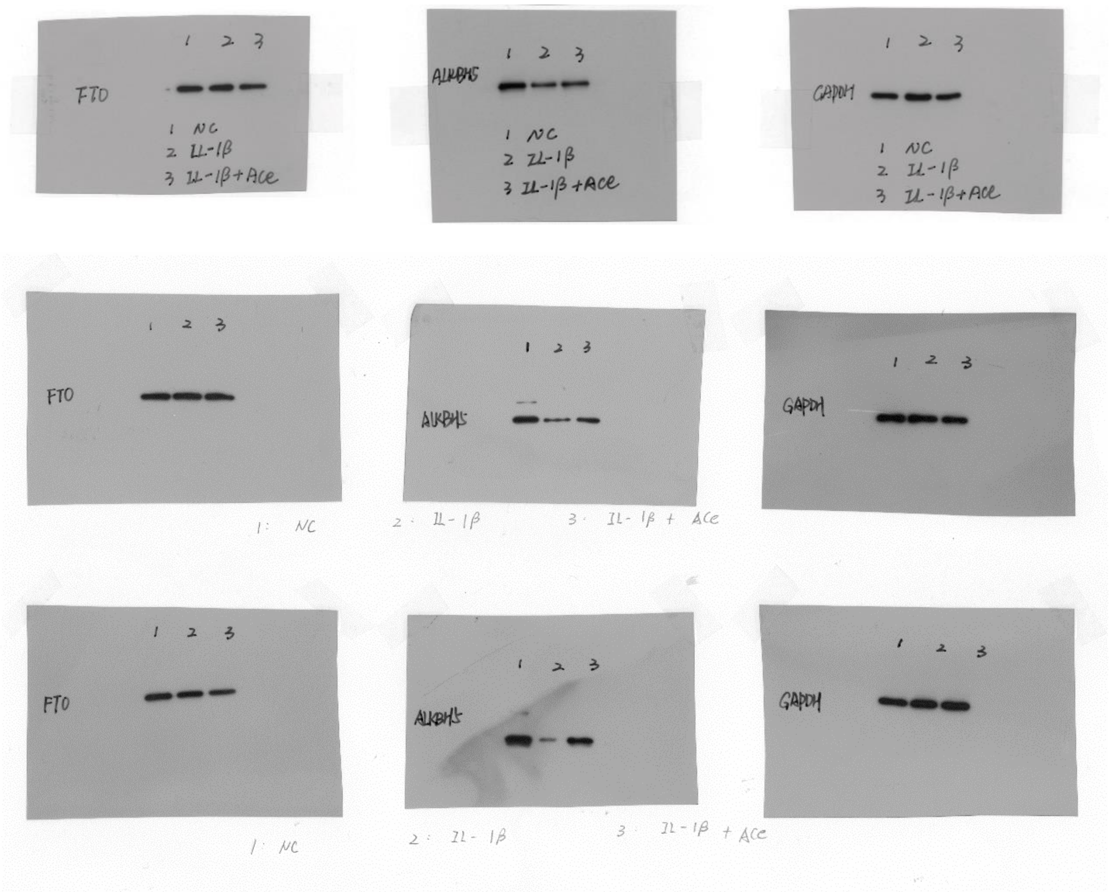

Supplementary Figure 3: The original versions of Figure 2D.

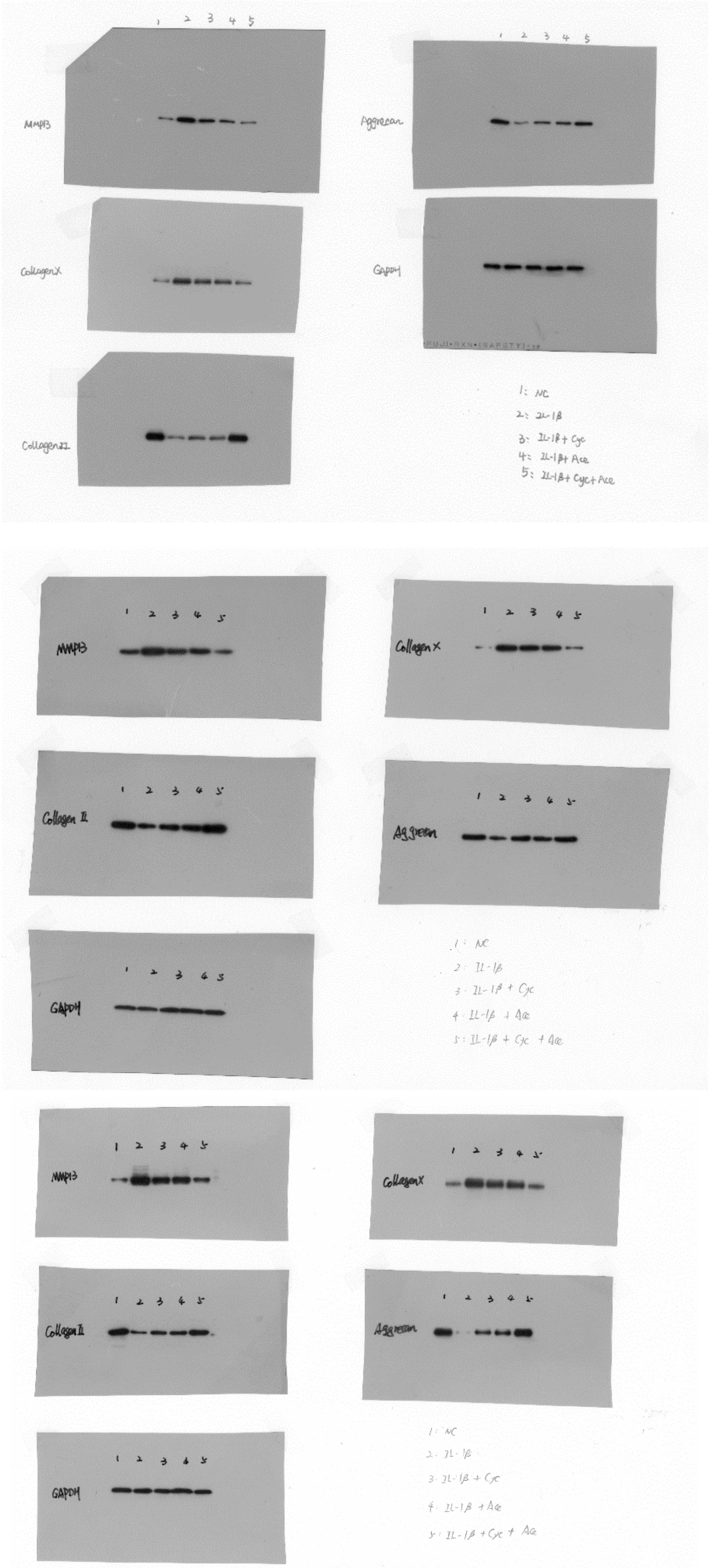

Supplementary Figure 4: The original versions of Figure 3A.

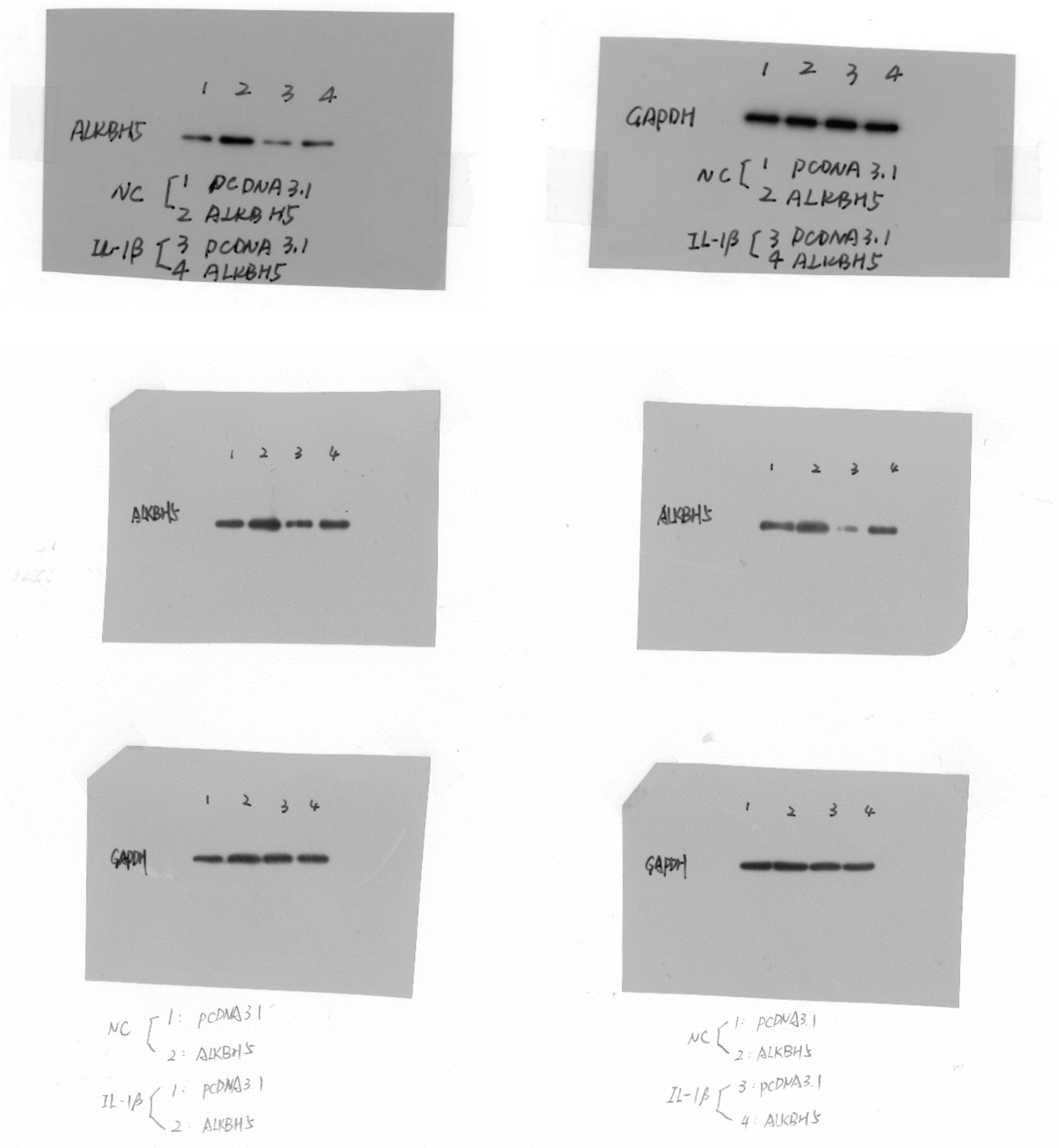

Supplementary Figure 5: The original versions of Figure 3E.

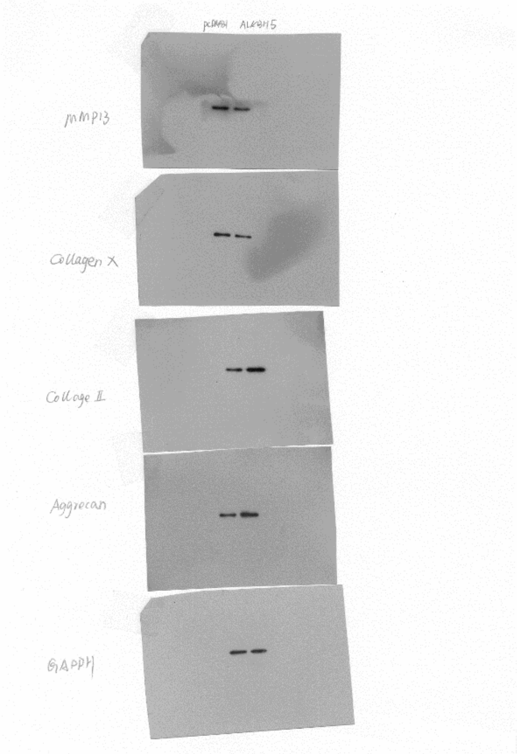

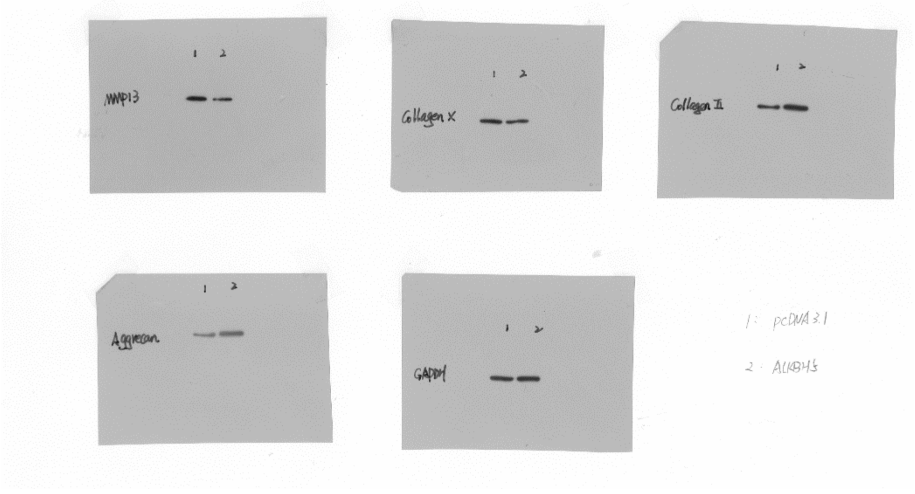

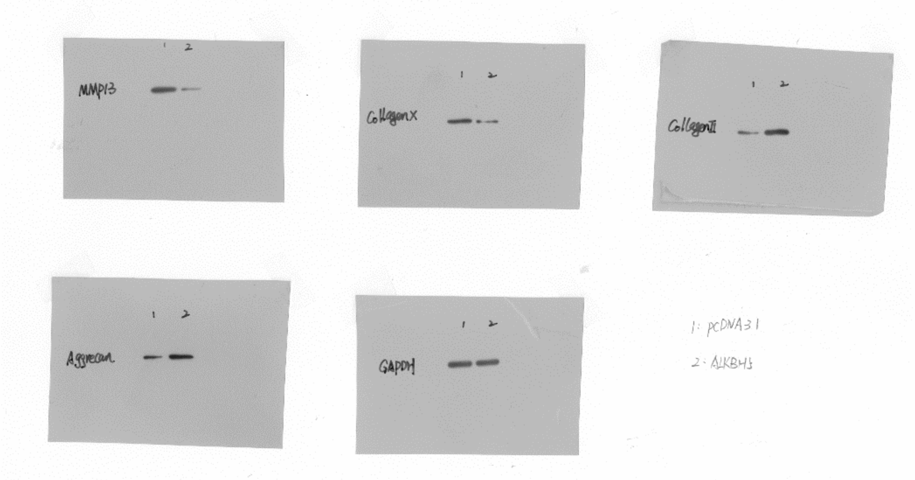

Supplement: Supplementary file 2 — Additional file 2: Supplementary Figure 2. The original versions of Fig. 1B. Supplementary Figure 3. The original versions of Fig. 2D. Supplementary Figure 4. The original versions of Fig. 3A. Supplementary Figure 5. The original versions of Fig. 3E. [file 12860_2022_444_MOESM2_ESM.pdf]
